# Epiploic Appendagitis in a Renal Transplant: A Case Report

**DOI:** 10.7759/cureus.29550

**Published:** 2022-09-25

**Authors:** Ghita El Bardai, Nada Jnyah, Basmat A Chouhani, Nadia Kabbali, Tarik Sqalli

**Affiliations:** 1 Nephrology, Dialysis, and Transplantation, Hassan II University Hospital, Sidi Mohamed Ben Abdellah University, Fez, MAR; 2 Nephrology, Dialysis, and Transplantation, Hassan II University Hospital, Fez, MAR

**Keywords:** diagnostic, primary epiploic appendagitis (pea), rare cause of acute abdominal pain, abdominal scan, kidney transplant

## Abstract

Appendagitis is an inflammation of the epiploic fringes, generally unrecognized by the clinician. It is responsible for abdominal pain and may mimic other causes of acute abdomen. It can be primary or secondary. In this article, we describe the first case of primary epiploic appendagitis in a renal transplant patient who consulted for left inguinoscrotal pain, which was diagnosed as primary epiploic appendagitis.

## Introduction

Epiploic appendagitis is an ischemic infarction of an epiploic appendage caused by torsion. It is considered a rare pathology for acute abdomen, occurring predominantly among males aged between 20 and 50 [1]. Obesity seems to be the only recognized risk factor [2]. This pathology has long been exceptionally diagnosed in the preoperative period. Advances in medical imaging have now enabled radiologists to make this diagnosis pre-operatively. Generally, it resolves spontaneously without surgery within a week or two. We describe the first case of primary omental appendagitis in a renal transplant recipient.

## Case presentation

The patient is a 23-year-old male, who followed up for end-stage chronic renal disease secondary to a segmental and focal hyalinosis, and started hemodialysis in 2017 by a left radio-radial arteriovenous fistula. He has no other medical or surgical history. He received a kidney transplant on April 22, 2022, from a living, related donor. The surgical procedure consisted of implantation of the graft in the right iliac fossa with end-to-side arterial and venous anastomosis on the external iliac and a Gregoir-type anastomosis with the temporary placement of a double J probe. The immunosuppressive therapy was based on tacrolimus, mycophenolate mofetil, and prednisone. The postoperative follow-up was uneventful.

He experienced three days of constant pain in the left inguinoscrotal area. Thirty-five days after the renal graft, he was feeling feverish without associated urinary or other digestive signs. The patient was apyretic, hemodynamically stable, with a BMI of 18. He had a sensitivity of the left iliac fossa with no palpable mass. The scar of the right graft was clean and painless, with no signs of inflammation. His complete blood count and C-reactive protein (CRP) were within normal range.

Abdominal and inguinoscrotal ultrasound was performed, showing a hypogastric formation compatible with a diagnosis of appendagitis, without testicular torsion or any abnormalities on the graft.

**Figure 1:**
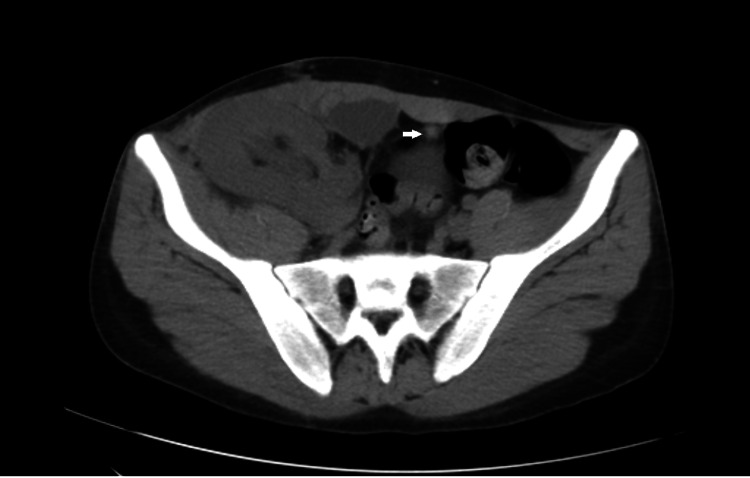
Abdominal scan without contrast in axial section showing the presence of an omental appendix twisted at the expense of sigmoid colon with infiltration of the surrounding fat evoking appendagitis; renal graft at right iliac fossa

**Figure 2:**
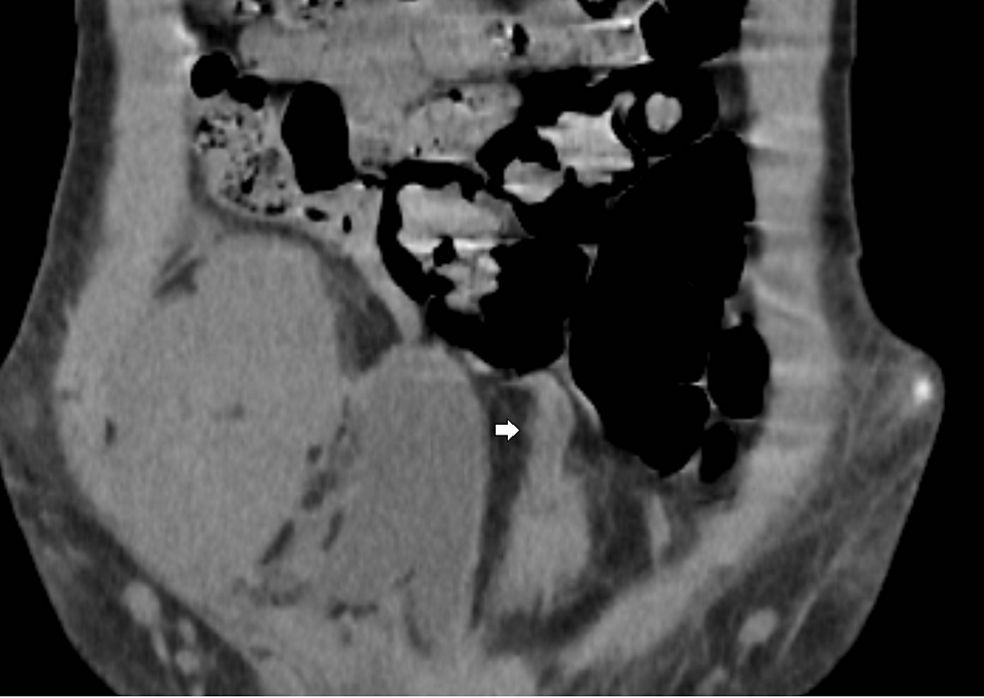
Abdominal scan without contrast in coronal section showing the presence of an omental appendix twisted at the expense of the sigmoid colon with infiltration of the surrounding fat-evoking appendagitis

**Figure 3:**
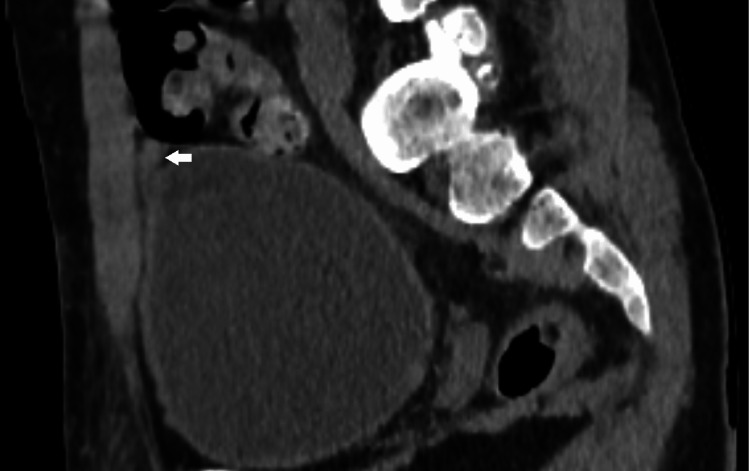
Abdominal scan without contrast in sagittal section showing the presence of an omental appendix twisted at the expense of sigmoid colon with infiltration of the surrounding fat-evoking appendagitis

The abdominal scan without injection revealed a hanging formation on the mesosigmoid, related to epiploic appendagitis of the sigmoid. It was then determined to be a primary epiploic appendagitis (Figures 1, 2, 3). The patient received paracetamol, codeine, and a combination of phloroglucinol/trimethyl, with a resolution of the symptoms within one week.

## Discussion

Epiploic appendagitis was first described in 1956 [1]. It is an inflammation of the epiploic appendages, which correspond to about 50 pedunculated fatty formations, measuring 1 to 2 cm in diameter and 0.5 to 5 cm in length, separated into two rows on the anterior and posterior sides of the colon [3]. They are located on the colonic frame but preferentially at the level of the rectosigmoid hinge (57%) and the ileocecal region (26%) [1]. They have a precarious vascularization, with only two arteries vascularizing each epiploic appendage. They also have a pedunculated morphology. These two characteristics favor inflammation. Primitive appendagitis is caused by thrombosis of the draining vein with or without torsion, or by inflammation without vascular abnormalities. It can also be secondary to pancreatitis, appendicitis, or any other intraperitoneal infection [3]. Clinically, the abdominal pain is constant and localized according to the location of the pathological appendage. In general, there is no abdominal guarding. Other digestive signs, such as nausea, vomiting, or transit disorders, are rare. The patient is sometimes subfebrile. In 10 to 30% of cases, a sub-parietal abdominal mass is palpable, its palpation causing extreme pain [3,4].

Contrary to the main differential diagnosis (sigmoid diverticulitis, appendicitis, pelvic inflammatory disease, etc.), the biological assessment is generally within normal limits. A slight elevation of the white blood count (WBC) and C-reactive protein (CRP) is rarely noted as an inflammatory response to fat necrosis [5].

The diagnosis used to be made during surgery. However, with the advancement of imaging, the diagnosis of appendagitis is made more easily by experienced radiologists. When the ultrasound is not very contributive, the abdominal scan with or without injection of iodinated contrast product enables the diagnosis by visualizing a "shuttle" image. It is an intra-peritoneal mass attached to the colon, the density of which is slightly higher than normal fat, often oval, well limited by a thin border of higher density, enhanced after injection of the iodinated contrast product, thus achieving a "ring sign" appearance [6,7].

The treatment is, in most cases, conservative, based on analgesic treatment, with or without non-steroidal anti-inflammatory drugs (NSAIDs), with the resolution of the pain in a week or two. Resection of the necrotic fringe is necessary in complicated cases, with good outcomes. The use of antibiotics does not seem justified, even in immunocompromised patients, unless there are signs of sepsis or bacteremia [1, 3, 5].

Appendagitis after transplantation was reported for the first time after liver transplantation for autoimmune hepatitis in a 71-year-old woman. Her treatment was based on mycophenolate mofetil, tacrolimus, and prednisone. An abdominal scan enabled the diagnosis. She was treated symptomatically with good evolution [8].

Is the occurrence of post-transplant appendagitis in our patient a simple coincidence? Or are there other factors, like surgery or immunosuppression, favoring its occurrence? To our knowledge, our case seems to be the first appendagitis occurring after renal transplantation. Other studies with a larger sample of patients are necessary to arrive at a conclusion.

## Conclusions

This case helps illustrate a rare and uncommon diagnosis of abdominal pain in renal transplant patients. In the case of acute abdominal pain without a major inflammatory syndrome, it is necessary to suspect appendagitis, among the other diagnoses. The confirmation is based on medical imaging to avoid unnecessary surgical interventions. Medical treatment with analgesics avoids an increase in the duration of hospitalization, as well as unnecessary antibiotic therapy.
